# Data from an International Multi-Centre Study of Statistics and Mathematics Anxieties and Related Variables in University Students (the SMARVUS Dataset)

**DOI:** 10.5334/jopd.80

**Published:** 2023-05-29

**Authors:** Jenny Terry, Robert M. Ross, Tamás Nagy, Mauricio Salgado, Patricia Garrido-Vásquez, Jacob O. Sarfo, Susan Cooper, Anke C. Buttner, Tiago J. S. Lima, İbrahim Öztürk, Nazlı Akay, Flavia H. Santos, Christina Artemenko, Lee T. Copping, Mahmoud M. Elsherif, Ilija Milovanović, Robert A. Cribbie, Marina G. Drushlyak, Katherine Swainston, Yiyun Shou, Juan David Leongómez, Nicola Palena, Fitri A. Abidin, Maria F. Reyes-Rodríguez, Yunfeng He, Juneman Abraham, Argiro Vatakis, Kristin Jankowsky, Stephanie N. L. Schmidt, Elise Grimm, Desirée González, Philipp Schmid, Roberto A. Ferreira, Dmitri Rozgonjuk, Neslihan Özhan, Patrick A. O’Connor, Andras N. Zsido, Gregor Stiglic, Darren Rhodes, Cristina Rodríguez, Ivan Ropovik, Violeta Enea, Ratri Nurwanti, Alejandro J. Estudillo, Nataly Beribisky, Karel K. Himawan, Linda M. Geven, Anne H. van Hoogmoed, Amélie Bret, Jodie E. Chapman, Udi Alter, Zoe M. Flack, Donncha Hanna, Mojtaba Soltanlou, Gabriel Banik, Matúš Adamkovič, Sanne H. G. van der Ven, Jochen A. Mosbacher, Hilal H. Şen, Joel R. Anderson, Michael Batashvili, Kristel de Groot, Matthew O. Parker, Mai Helmy, Mariia M. Ostroha, Katie A. Gilligan-Lee, Felix O. Egara, Martin J. Barwood, Karuna Thomas, Grace McMahon, Siobhán M. Griffin, Hans-Christoph Nuerk, Alyssa Counsell, Oliver Lindemann, Dirk Van Rooy, Theresa E. Wege, Joanna E. Lewis, Balazs Aczel, Conal Monaghan, Ali H. Al-Hoorie, Julia F. Huber, Saadet Yapan, Mauricio E. Garrido Vásquez, Antonino Callea, Tolga Ergiyen, James M. Clay, Gaetan Mertens, Feyza Topçu, Merve G. Tutlu, Karin Täht, Kristel Mikkor, Letizia Caso, Alexander Karner, Maxine M. C. Storm, Gabriella Daroczy, Rizqy A. Zein, Andrea Greco, Erin M. Buchanan, Katharina Schmid, Thomas E. Hunt, Jonas De keersmaecker, Peter E. Branney, Jordan Randell, Oliver J. Clark, Crystal N. Steltenpohl, Bhasker Malu, Burcuş Teke, TamilSelvan Ramis, Stefan Agrigoroaei, Nicholas A. Badcock, Kareena McAloney-Kocaman, Olena V. Semenikhina, Erich W. Graf, Charlie Lea, Kalu T. U. Ogba, Fergus M. Guppy, Amy C. Warhurst, Shane Lindsay, Ahmed Al Khateeb, Frank Scharnowski, Leontien de Kwaadsteniet, Kathryn B. Francis, Mariah Lecompte, Lisa A. D. Webster, Kinga Morsanyi, Suzanna E. Forwood, Elizabeth R. Walters, Linda K. Tip, Jordan R. Wagge, Ho Yan Lai, Deborah S. Crossland, Kohinoor M. Darda, Tessa R. Flack, Zoe Leviston, Matthew Brolly, Samuel P. Hills, Elizabeth Collins, Andrew J. Roberts, Wing-Yee Cheung, Sophie Leonard, Bruno Verschuere, Samantha K. Stanley, Iro Xenidou-Dervou, Omid Ghasemi, Timothy Liew, Daniel Ansari, Johnrev Guilaran, Samuel G. Penny, Julia Bahnmueller, Christopher J. Hand, Unita W. Rahajeng, Dar Peterburg, Zsofia K. Takacs, Michael J. Platow, Andy P. Field

**Affiliations:** 1 School of Psychology, University of Sussex, UK; 2 Department of Philosophy, Macquarie University, Australia; 3 Institute of Psychology, ELTE Eötvös Loránd University, Hungary; 4 School of Social Sciences, Universidad Andres Bello, Chile; 5 Centre for Research in Inclusive Education, Universidad Andres Bello, Chile; 6 Department of Psychology, University of Concepción, Chile; 7 Department of Health, Physical Education, and Recreation, University of Cape Coast, Ghana; 8 Department of Psychology, Kingston University, UK; 9 School of Psychology, University of Birmingham, UK; 10 Department of Social and Work Psychology, University of Brasília, Brazil; 11 Department of Psychology, Middle East Technical University, Turkey; 12 School of Psychology, University College Dublin, Ireland; 13 Department of Psychology, University of Tuebingen, Germany; 14 Department of Psychology, Teesside University, UK; 15 Department of Psychology, Faculty of Philosophy, University of Novi Sad, Serbia; 16 Department of Psychology, York University, Canada; 17 Mathematics Department, Makarenko Sumy State Pedagogical University, Ukraine; 18 School of Psychology, Newcastle University, UK; 19 Research School of Psychology, Australian National University, Australia; 20 Saw Swee Hock School of Public Health, National University of Singapore, Singapore; 21 Faculty of Psychology, Universidad El Bosque, Colombia; 22 Department of Human and Social Sciences, University of Bergamo, Italy; 23 Faculty of Psychology, Universitas Padjadjaran, Indonesia; 24 Department of Psychology, Universidad de los Andes, Colombia; 25 Liaoning Key Laboratory of Psychological Testing and Behavior Analysis, Liaoning Univeristy, China; 26 Department of Psychology, Faculty of Humanities, Bina Nusantara University, Indonesia; 27 Department of Psychology, Panteion University of Social and Political Sciences, Greece; 28 Psychological Assessment, University of Kassel, Germany; 29 Department of Psychology, University of Konstanz, Germany; 30 Psychological Sciences Research Institute, UCLouvain, Belgium; 31 Departamento de Didáctica e Investigación Educativa, Universidad de la Laguna, Spain; 32 Media and Communication Science, University of Erfurt, Germany; 33 Implementation Research, Bernhard-Nocht-Insitute for Tropical Medicine, Germany; 34 Facultad de Ciencias de la Educación, Universidad Católica del Maule, Chile; 35 Millennium Nucleus for the Science of Learning (MiNSoL), Chile; 36 Institute of Mathematics and Statistics, University of Tartu, Estonia; 37 Department of Molecular Psychology, Ulm University, Germany; 38 School of Psychology, University of Southampton, UK; 39 School of Psychology, Queen’s University Belfast, UK; 40 Institute of Psychology, University of Pécs, Hungary; 41 Faculty of Health Sciences, University of Maribor, Slovenia; 42 Usher Institute, University of Edinburgh, UK; 43 NTU Psychology, Nottingham Trent University, UK; 44 Charles University, Faculty of Education, Institute for Research and Development of Education, Czech Republic Faculty of Education, University of Presov, Slovakia; 45 Department of Psychology, Alexandru Ioan Cuza University, Romania; 46 Department of Psychology, Brawijaya University, Indonesia; 47 Department of Psychology, Bournemouth University, UK; 48 School of Psychology, University of Nottingham, Malaysia; 49 Faculty of Psychology, Universitas Pelita Harapan, Indonesia; 50 RELASI Research Lab, Indonesia; 51 Institute of Criminal Law and Criminology, Leiden University, the Netherlands; 52 Department of Clinical Psychology, University of Amsterdam, the Netherlands; 53 Behavioural Science Institute, Radboud University, the Netherlands; 54 LPPL, Nantes University, France; 55 School of Psychology, Australian Catholic University, Australia; 56 Department of Psychology, Toronto Metropolitan University, Canada; 57 School of Humanities and Social Science, University of Brighton, UK; 58 School of Psychology, University of Surrey, UK; 59 Brain and Mind Institute & Department of Psychology, Western University, Canada; 60 University of Presov, Slovakia; 61 Institute of Social Sciences, CSPS, Slovak Academy of Sciences, Slovakia; 62 Faculty of Humanities and Social Sciences, University of Jyväskylä, Finland; 63 Institute of Psychology, University of Graz, Austria; 64 Department of Psychology, MEF University, Turkey; 65 Faculty of Psychology, University of Akureyri, Iceland; 66 Australian Research Centre in Sex, Health and Society, Australia; 67 Baruch Ivcher School of Psychology, Reichman University, Israel; 68 Department of Psychology, Erasmus School of Social and Behavioural Sciences, Erasmus University Rotterdam, the Netherlands; 69 Department of Applied Economics, Erasmus School of Economics, Erasmus University Rotterdam, the Netherlands; 70 School of Pharmacy and Biomedical Science, University of Portsmouth, UK; 71 Department of Psychology, College of Education, Sultan Qaboos University, Oman; 72 Department of Psychology, Faculty of Arts, Menoufia University, Egypt; 73 Computer Science Department, Makarenko Sumy State Pedagogical University, Ukraine; 74 Department of Science Education, University of Nigeria, Nsukka; 75 School of Health, Sport, and Life Sciences, Leeds Trinity University, UK; 76 Department of Psychology, HELP University, Malaysia; 77 Department of Psychology, University of Limerick, Ireland; 78 Institute for Climate, Energy, and Disaster Solutions, Australia; 79 Centre for Mathematical Cognition, Loughborough University, UK; 80 University of Northern Colorado, USA; 81 Independent Researcher, Saudi Arabia; 82 Department of Psychology, Hasan Kalyoncu University, Turkey; 83 Department of Human Sciences, Libera Università Maria SS. Assunta University, Italy; 84 Izmir University of Economics, Turkey; 85 Department of Psychology, University of Portsmouth, UK; 86 Department of Medical and Clinical Psychology, Tilburg University, the Netherlands; 87 Institute of Psychology, University of Tartu, Estonia; 88 Department of Cognition, Emotion and Methods in Psychology, University of Vienna, Austria; 89 Department of Psychology, Universitas Airlangga, Indonesia; 90 Harrisburg University of Science and Technology, USA; 91 Universitat Ramon Llull, Esade Business School, Spain; 92 School of Psychology, University of Derby, UK; 93 Ramon Llull University, Esade Business School, Spain; 94 Department of Psychology, University of Bradford, UK; 95 Department of Psychology, University of Winchester, UK; 96 Department of Psychology, Manchester Metropolitan University, UK; 97 University of Southern Indiana, USA; 98 Dartmouth Center for Program Design and Evaluation, USA; 99 O P Jindal Global University, India; 100 Department of Psychology, Başkent University, Turkey; 101 Centre for American Education, Sunway University, Malaysia; 102 School of Psychological Science, University of Western Australia, Australia; 103 School of Psychological Sciences, Macquarie University, Australia; 104 Department of Psychology, Glasgow Caledonian University, UK; 105 Computer Science Depatment, Makarenko Sumy State Pedagogical University, Ukraine; 106 Psychology Department, University of Nigeria, Nigeria; 107 School of Applied Sciences, University of Brighton, UK; 108 School of Energy, Geoscience, Infrastructure and Society, Heriot-Watt University, UK; 109 School of Psychology, University of Winchester, UK; 110 Department of Psychology and Social Work, University of Hull, UK; 111 Psychiatric Hospital, University of Zürich, Switzerland; 112 School of Psychology, Keele University, UK; 113 School of Psychology and Therapeutic Studies, Leeds Trinity University, UK; 114 School of Psychology & Sport Science, Anglia Ruskin University, UK; 115 School of Psychology and Cognitive Science, Avila University, USA; 116 University of Pennsylvania, USA; 117 School of Psychology, University of Lincoln, UK; 118 School of Arts & Humanities, Edith Cowan University, Perth, WA, Australia; 119 Faculty of Health and Social Sciences, Bournemouth University, UK; 120 Division of Psychology, Faculty of Natural Sciences, University of Stirling, UK; 121 School of Psychology, University of New South Wales, Australia; 122 Department of Psychology, Western University, Canada; 123 Division of Social Sciences, College of Arts and Sciences, University of the Philippines Visayas, Philippines; 124 Bristol Zoological Society, Bristol, UK; 125 School of Education, University of Glasgow, UK; 126 School of Health in Social Science, University of Edinburgh, UK

**Keywords:** Statistics, mathematics, anxiety, education, jangle fallacy

## Abstract

This large, international dataset contains survey responses from *N* = 12,570 students from 100 universities in 35 countries, collected in 21 languages. We measured anxieties (statistics, mathematics, test, trait, social interaction, performance, creativity, intolerance of uncertainty, and fear of negative evaluation), self-efficacy, persistence, and the cognitive reflection test, and collected demographics, previous mathematics grades, self-reported and official statistics grades, and statistics module details. Data reuse potential is broad, including testing links between anxieties and statistics/mathematics education factors, and examining instruments’ psychometric properties across different languages and contexts. Data and metadata are stored on the Open Science Framework website [https://osf.io/mhg94/].

## (1) Background

Many university students on non-mathematics-based degrees report feeling anxious about learning mathematics and statistics (e.g., Field, 2014). *Statistics anxiety* was initially assumed to be the same as *mathematics anxiety*, but many now consider it distinct (Chew & Dillon, 2014). Statistics anxiety has been defined as “a negative state of emotional arousal experienced by individuals as a result of encountering statistics in any form and at any level […] and is related to but distinct from mathematics anxiety” (Chew & Dillon, 2014, p. 199). Mathematics anxiety is similarly defined as involving “feelings of tension and anxiety that interfere with the manipulation of numbers and the solving of mathematical problems in […] ordinary life and academic situations” (Richardson & Suinn, 1972, p. 551). Neither definition is clear about how these two constructs differ, and students may perceive them to be the same because both mathematics and statistics involve the manipulation and interpretation of numerical information. This conflation could be a shared root of students’ anxiety, rather than their anxiety being specific to mathematics or statistics.

These definitions have informed the scales that measure these constructs (Baloğlu & Zelhart, 2007; Cruise et al., 1985). For these scales to be valid, we need clarity about whether they measure facets of anxiety specific to statistics/mathematics or reflect a common *numeric anxiety*. In short, we must rule out a *jangle fallacy*, where two scales are incorrectly assumed to measure different constructs (Kelley, 1927). Jangle fallacies can lead to independently evolving theoretical literatures for each construct that should instead be mutually informative (Block, 1995).

Few studies have tested the distinctiveness of statistics and mathematics anxiety scales. Most concluded statistics anxiety is related to mathematics anxiety, but some variance remains unaccounted for, suggesting a unique component (*r* = 0.41 to *r* = 0.67; Baloğlu, 2002; Birenbaum & Eylath, 1994; Paechter et al., 2017; Zeidner, 1991). What this unique component is remains unclear. It is possible the unexplained variance does not reflect differences in statistics and mathematics anxieties, but differences in the scales’ dimensions. For example, because the Statistics Anxiety Rating Scale (STARS; Cruise et al., 1985) includes a “fear of asking for help” subscale and the Revised Maths Anxiety Rating Scale (R-MARS; Baloğlu & Zelhart, 2007) does not, the unique variance may have been driven by the fear of asking for help only captured by the STARS.

It is important to use a range of methods to study the constructs’ independence, such as various confirmatory factor analysis techniques (Lawson & Robins, 2021), extrinsic convergent validity analysis (Gonzalez et al., 2020), and multi-trait-multi-method designs (Campbell & Fiske, 1959). However, previous studies that compared measures of mathematics and statistics anxiety (e.g., Baloğlu, 2002; Paechter et al., 2017) have based their conclusions on correlations, which are only one of the 10 criteria that can determine the extent that two scales overlap (Lawson & Robins, 2021).

To address these concerns, Terry et al. (2023) explored these constructs’ distinctiveness in two samples of UK-based undergraduate psychology students (*N* = 465 and *N* = 245). They measured statistics anxiety with the STARS (Cruise et al., 1985) and mathematics anxiety with the R-MARS (Baloğlu & Zelhart, 2007), and developed versions of each scale modified to reflect the other construct (i.e., a mathematics version of the STARS and a statistics version of the R-MARS). By doing so, Terry et al. (2023) created equivalent, comparable subscales (e.g., there was now a mathematics version of the “Fear of asking for help” subscale). Their results suggested a jangle fallacy. Specifically, the factor analyses and latent profile analyses of the four measures, as well as their experimental studies, found converging evidence that the scales were measuring the same construct.

However, construct validation work should be conducted for all populations that use a given measure (Flake, 2021) and with statistics being a required module^1^ for undergraduate students of most social and physical sciences in universities throughout the world (Schwab-McCoy, 2019), the extent to which these findings are generalisable should be examined.

Therefore, the first aim of the present study was to assess generalisability by repeating Terry et al.’s (2023) study in a large, international sample of university students from different academic disciplines for whom statistics was part of their degrees.

Our second aim was to explore whether specific facets of the STARS and R-MARS are driven by a superordinate *parent construct* (Lawson & Robins, 2021). For example, scores on the scales’ test anxiety items might be driven by general test anxiety, and not specific to mathematics or statistics tests. Therefore, we added further measures of *fear of negative evaluation* (Carleton et al., 2011), *intolerance of uncertainty* (Carleton et al., 2007), *social interaction* and *performance anxiety* (Baker et al., 2002; Liebowitz, 1987), *creativity anxiety* (Daker et al., 2020), *test anxiety* (Benson & El-Zahhar, 1994), and *trait anxiety* (Ree et al., 2008) to assess whether they underpin STARS and R-MARS items.

Our third aim was to examine the constructs’ extrinsic convergent validity (ECV; the extent two measures correlate with other constructs in the same ways; Gonzalez et al., 2020). The more similar the correlations are, the more probable it is that the measures are tapping the same construct (Gonzalez et al., 2020). To examine ECV, we included five additional variables shown to correlate with statistics and/or mathematics anxieties: *Self-efficacy* (e.g., *Z* = |0.52|; Trassi et al., 2022), *persistence* (e.g., *r* = –.75; González et al., 2016), *analytic thinking* (using a revised version of the Cognitive Reflection Test; CRT; Shenhav et al., 2012),^2^ pre-university mathematics qualifications (e.g., *r* = –.27; Beurze et al., 2013), and university statistics module grades (although, this relationship varies from *r* = –.56 to *r* = .10; Terry & Field, 2023).

Besides our core aims, we maximised the reuse potential of this dataset – particularly its capacity to address important questions in the statistics education literature (see Section 4 – Reuse Potential) – by collecting data from the student participants’ statistics instructors about their module format, content, and assessment.

## (2) Methods

### 2.1 Study design

The data were collected via a cross-sectional, online, self-report questionnaire-based, multi-centre study. The final dataset was generated from the following three sources (see Section 2.5 for full details of each variable):

1. The *student survey*, containing survey responses from university students on measures of statistics and mathematics anxieties (including the modified versions from Terry et al., 2023), test anxiety, trait anxiety, fear of negative evaluation, social interaction anxiety and performance anxiety, intolerance of uncertainty, creativity anxiety, self-efficacy, persistence, analytical thinking, and belief in God/s. Students also provided demographic information (age, gender/sex, ethnicity, and any specific learning difficulties), information about their pre-university mathematics qualifications (highest level, grades, and how long ago they were taken), self-reported grades for completed statistics modules, and their degree course details (university, major, year of study, and whether they are studying any non-statistics mathematics-based modules). Student survey data also include selected information auto-recorded by Qualtrics (Qualtrics, Provo, UT; the start and end dates, duration, and completion percentage for each response) and key identifiers added by the lead author (participant ID, survey ID, country, and language).
2. An *instructor survey*, containing information about the statistics modules students were taking at the time of completing the survey. The instructor survey recorded dates of the student participants’ statistics module, mode of teaching delivery (e.g., lectures/workshops, online/face-to-face), module content, and types and dates of assessments. Instructors also indicated how assessments were graded, necessary to standardise grades across institutions.
3. Students’ *grade data* from university records (where permitted to obtain and share).

### 2.2 Time of data collection

Data were collected between January 2021 and September 2021.^3^ Due to the differences in term/semester dates cross-nationally, different research teams had different start and end dates. The date participants began and finished the survey is included in the dataset.

### 2.3 Location of data collection

### 2.4 Sampling, sample and data collection

Collaborating research teams were recruited via Twitter and word-of-mouth, with efforts made to invite researchers from geographically and culturally diverse countries with varying education systems to produce more generalisable results. In the end, data were collected from 100 universities in 35 countries and in 21 languages.

#### Student Survey

Convenience sampling was used to recruit student participants. Most students (at least 80.4%)^5^ were invited to take part by collaborating researchers (or those with access to the sample on researchers’ behalf, such as statistics module instructors) via email, virtual learning environments, university-specific student social media platforms, and university participant pools^6^ and took part in their own time. Some students (at least 2.9%) were invited to complete the survey as part of an in-class exercise. Participation was always voluntary, and students were able to withdraw during the study and up to four weeks after taking part. The study was hosted via Qualtrics online survey software (Qualtrics, Provo, UT) and students completed it using a suitable electronic device (e.g., laptop, mobile phone, or tablet)^7^ with internet access.

A total of *N* = 18,841 student survey responses were recorded. For the present version of the data, we have excluded any cases where the participant began the survey but withdrew before the first block of measurement scale items; *n* = 6,199) and any duplicates (*n* = 72), which were identified using a combination of participant-generated ID code and demographic responses. In line with our pre-registration (https://osf.io/xs5wf), the case with the most complete data was retained, or, if both cases contained the same amount of data, the case with the earliest start date (i.e., the participant’s first attempt) was retained. In line with our goal to provide the data in as raw a form as possible, we have not excluded any other data. Note that for our primary research study, we planned to recruit undergraduate students that had taken or were taking statistics as part of their research methods training on any degree course that is not typically associated with mathematics. For example, we would exclude courses such as physics, engineering, and data science, whilst courses such as social sciences, business, and geography were eligible. Despite this stipulation, some responses were received from postgraduate students (*n* = 3), and from those on mathematics and statistics degrees (*n* = 2), mathematics-adjacent degrees (e.g., physics, engineering, computer sciences; *n* = 151), and degrees that are unlikely to have included a statistics module (e.g., arts & humanities subjects; *n* = 232). We have included these responses in the present dataset to afford other researchers the opportunity to set their own exclusion criteria. Similarly, the pre-registration for the primary empirical study (https://osf.io/xs5wf) states that we would only retain responses that passed the attention checks, but we have not removed them in the present data (*n* = 8,597 responded to and passed all seven; see Quality Control for more details).

**Figure 1:**
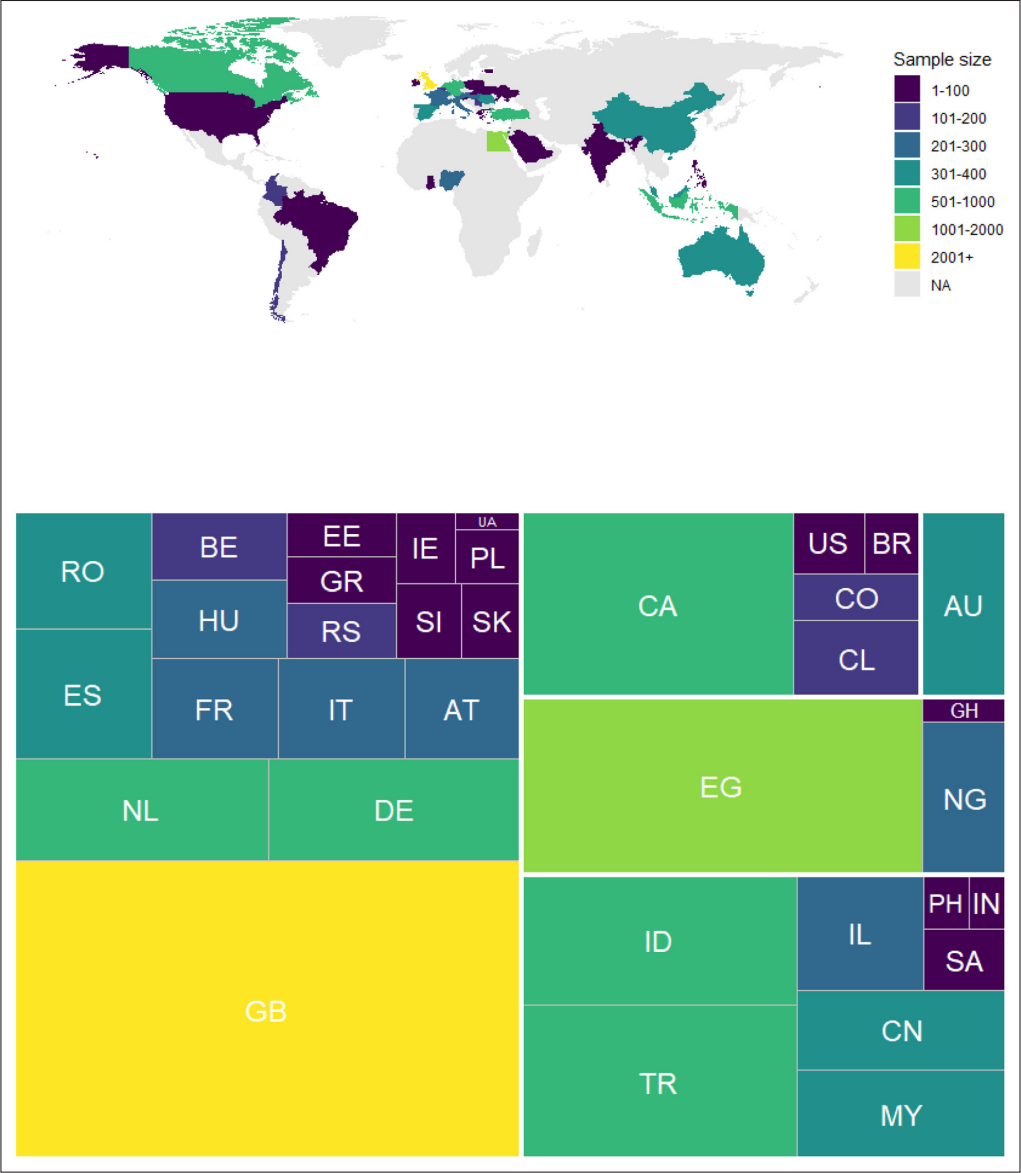
The top panel is a map showing the countries from which data were collected and their respective sample sizes. The bottom panel is a treemap of sample sizes for each country, organised by continents (see Table 1 for ISO country codes).

After exclusions, the final sample presented here contains *n* = 12,570 responses (68.2% of initial responses). Table 1 contains a breakdown of the number of responses from each university and Figure 1 offers a visual summary of how sample sizes varied worldwide.

**Table 1:** A table detailing the universities data were collected from, the country they were in,^4^ associated survey language, and the number of responses at the country and university level (after exclusions).


COUNTRY (ISO CODE; *N*)	LANGUAGE	UNIVERSITY	*N*

Australia (AU; *N* = 315)			

	English	Macquarie University	237

	English	Australian National University	53

	English	University of Western Australia	25

Austria (AT; *N* = 230)			

	German	University of Vienna	120

	German	University of Graz	108

	German	Medical University of Graz	1

	German	Technical University of Graz	1

Belgium (BE; *N* = 184)			

	French	Catholic University of Louvain (UCLouvain)	184

Brazil (BR; *N* = 68)			

	Portuguese	University of Brasília	58

	Portuguese	UNESP – São Paulo State University	10

Canada (CA; *N* = 986)			

	English	Toronto Metropolitan University (formerly Ryerson University)	520

	English	York University	228

	English	Memorial University of Newfoundland	127

	English	Western University	111

Chile (CL; *N* = 191)			

	Spanish	Andrés Bello National University	98

	Spanish	University of Concepción	93

China (CN; *N* = 323)			

	Chinese	Tianjin Normal University	196

	Chinese	Qufu Normal University	127

Colombia (CO; *N* = 114)			

	Spanish	El Bosque University	113

	Spanish	Other (unspecified)	1

Egypt (EG; *N* = 1,390)			

	Arabic	Menoufia University	1,390

Estonia (EE; *N* = 98)			

	Estonian	University of Tartu	91

	Estonian	Tallinn University	7

France (FR; *N* = 248)			

	French	University of Nantes	248

Germany (DE; *N* = 506)			

	German	University of Erfurt	231

	German	University of Konstanz	114

	German	University of Tübingen	110

	German	University of Kassel	50

	German	International University of Applied Sciences	1

Ghana (GH; *N* = 41)			

	English	University of Education, Winneba	19

	English	University of Cape Coast	9

	English	University of Ghana	7

	English	All Nations University	2

	English	Kwame Nkrumah University of Science and Technology	1

	English	Other (unspecified)	1

	English	University of Health and Allied Sciences	1

	English	University of Professional Studies, Accra	1

Greece (GR; *N* = 99)			

	Greek	Panteion University	94

	Greek	Aristotle University of Thessaloniki	2

	Greek	National and Kapodistrian University of Athens	2

	Greek	University of Crete	1

Hungary (HU; *N* = 206)			

	Hungarian	ELTE Eötvös Loránd University	184

	Hungarian	University of Pécs	22

India (IN; *N* = 41)			

	English	CHRIST (deemed to be) University	41

Indonesia (ID; *N* = 697)			

	Bahasa Indonesia	Bina Nusantara University	223

	Bahasa Indonesia	Brawijaya University	171

	Bahasa Indonesia	Airlangga University	131

	Bahasa Indonesia	Pelita Harapan University	96

	Bahasa Indonesia	Padjadjaran University	62

	Bahasa Indonesia	Atma Jaya Catholic University of Indonesia	14

Ireland (IE; *N* = 82)			

	English	University of Limerick	60

	English	University College Dublin	22

Israel (IL; *N* = 285)			

	Hebrew	Reichman University (née Interdisciplinary Center Herzliya)	285

Italy (IT; *N* = 248)			

	Italian	University of Bergamo	176

	Italian	LUMSA University	72

Malaysia (MY; *N* = 369)			

	English	HELP University	369

Netherlands (NL; *N* = 508)			

	Dutch	Radboud University	165

	Dutch	Tilburg University	133

	English	University of Amsterdam	114

	Dutch	Erasmus University Rotterdam	96

Nigeria (NG; *N* = 255)			

	English	University of Nigeria	255

Philippines (PH; *N* = 47)			

	English	University of the Philippines Visayas	47

Poland (PO; *N* = 69)			

	Polish	University of Silesia	58

	Polish	WSB University, Poznan	11

Romania (RO; *N* = 317)			

	Romanian	Alexandru Ioan Cuza University	317

Saudi Arabia (SA; *N* = 100)			

	Arabic	King Faisal University	100

Serbia (RS; *N* = 117)			

	Serbian	University of Novi Sad	117

Slovakia (SL; *N* = 88)			

	Slovakian	University of Prešov	88

Slovenia (SL; *N* = 94)			

	Slovenian	University of Maribor	94

Spain (ES; *N* = 346)			

	Spanish	University of La Laguna	218

	English	ESADE Business School, Universitat Ramon Llull	128

Turkey (TR; *N* = 834)			

	Turkish	Hasan Kalyoncu University	339

	Turkish	MEF University	160

	Turkish	Baskent University	158

	Turkish	Izmir University of Economics	100

	Turkish	Middle East Technical University	77

UK (GB; *N* = 2,962)			

	English	University of Sussex	413

	English	University of Birmingham	363

	English	Bournemouth University	214

	English	Nottingham Trent University	202

	English	University of Southampton	163

	English	Kingston University	157

	English	Queen’s University Belfast	137

	English	Loughborough University	134

	English	University of Stirling	125

	English	University of Lincoln	124

	English	University of Hull	123

	English	University of Portsmouth	116

	English	University of Winchester	107

	English	University of Brighton	99

	English	University of Surrey	99

	English	Teesside University	90

	English	University of Derby	90

	English	Glasgow Caledonian University	60

	English	University of Bradford	56

	English	Anglia Ruskin University	36

	English	Manchester Metropolitan University	32

	English	Leeds Trinity University	22

Ukraine (UA; *N* = 25)			

	Ukrainian	Sumy Makarenko State Pedagogical University	25

USA (US; *N* = 87)			

	English	University of Southern Indiana	51

	English	University of Northern Colorado	33

	English	Avila University	3

Total			12,570


Participants’ ages ranged from 18 to 67 years (*M* = 21.01, *SD* = 4.12); with 3,119 participants choosing not to respond to this question and 14 values (≥99 years) recoded as implausible. The majority of participants identified as^8^ a woman/female (*n* = 8,298, 66.0%), with a further 2,002 identifying as a man/male (15.9%), 74 as non-binary (0.6%), 13 preferred to describe their gender in another way (0.1%),^9^ and 2,183 (17.4%) did not respond to this question. Most participants (*n* = 9,026, 71.8%) reported they did not have a diagnosis of any of the following Specific Learning Differences (SpLDs): ADHD/ADD, Dyslexia, Dyscalculia, Dyspraxia, or Dysgraphia/Dysorthography. However, 738 (5.9%) participants reported having one or more SpLD, whilst a further 111 (0.8%) responded “other” (including self-diagnosis), 3 were unsure (<0.1%), and 2,692 (21.4%) did not respond.

Most participants indicated they were in the first year of their degree course (*n* = 4,505, 35.8%), with a further 3,126 in second year (24.9%), 1,859 in third year (14.8%), 689 in fourth year (5.5%), and 40 in fifth year (0.3%). An additional 61 participants (0.5%) indicated their degree year as ‘other’, three participants (0.02%) were postgraduates, and 2,287 (18.2%) did not respond. Psychology was the most common degree major amongst participants (*n* = 8,759, 69.7%), followed by Business and Finance (*n* = 768, 6.1%), Education (*n* = 397, 3.2%), Health and Medical Sciences (*n* = 273, 2.2%), and Computer Sciences (*n* = 128, 1.0%).^10^ A further 1,526 (12.1%) of students did not indicate their degree major.

Each university provided their own participation incentives based on local norms and availability. Half of participants were offered ungraded course credits (50.0%) and around a third were offered no incentive (33.0%), with the remaining being offered either a prize draw (up to a maximum of £50 or local equivalent per 100 participants; 10.0%), payment (maximum £5 or local equivalent; 3.1%), a choice of a prize draw or course credits (3.3%), or both payment and course credits (0.5%). Incentive information is unavailable for 0.1% of participants.

#### Instructor Survey

The student participants’ statistics module instructors were invited to take part by email (either by the lead researcher, where the collaborating researchers were also module instructors, or by the collaborating researchers where there were not). In some cases, someone other than the primary instructor completed the survey (e.g., graduate teaching assistants). Participation was voluntary and not a condition of involvement with the project. The instructor survey was also hosted via Qualtrics online survey software (Qualtrics, Provo, UT).

A total of *N* = 176 instructor survey responses were recorded. We have excluded responses given in error (e.g., for a postgraduate course or for more than one module per response; *n* = 21) and any cases where no data was entered (*n* = 36).

After exclusions, the final sample contained *n* = 119 responses (67.6% of initial responses), representing *n* = 96 modules in *n* = 57 universities in *n* = 27 countries, corresponding to *n* = 4,867 student survey responses.

#### Grade Data

Where permitted by the student participants and by their universities, we also collected grades (and grading scales) for the statistics module students were taking at the time of completing the survey from university records.^11^ A total of *N* = 20 universities provided this data, corresponding to *n* = 1,804 student participants in *n* = 41 modules in *n* = 9 countries.

### 2.5 Materials/Survey instruments

#### Survey Adaptations

The student survey was prepared in stages. First, a generic master version of the survey was created in English by the lead researcher (available here: https://osf.io/enc29). This version was then adapted from English into the local language by collaborating research teams as required, resulting in a generic master version for each language. A translation guide was provided (available here: https://osf.io/v3qxf), which advised translators to adopt a team-based approach (Behr & Shishido, 2016). This approach involved a minimum of two people translating the scales individually and resolving any differences as a team. It was chosen over the more widespread back-translation technique, because it is more effective in producing equivalent scales across languages (Behr, 2017).

The generic master version for each language was then copied for each research team for modification to the local context, following guidelines provided to encourage consistency (available here: https://osf.io/t2pc5). Modifications were kept minimal and primarily pertained to course/module details (e.g., the names of the statistics modules), the mathematics education questions (e.g., to reflect the structure of pre-university education locally), and the demographic questions (e.g., adapting the ethnicity options to reflect local populations). Researchers could also adapt it to award participant incentives (e.g., linking to local course credit systems). The measurement scales were not altered, with minor exceptions (detailed in the Measures section below). Very rarely, and where it did not impact our core research aims, questions were removed altogether to meet the requirements of the local ethics boards and/or to be appropriate in the local context (e.g., some ethics boards requested we did not ask about ethnicity). Data that are missing from the student survey because a question was omitted is recorded in the data as ‘Not Administered’.

The instructor survey was not adapted, and all respondents took the same English-language version of the survey.

All materials, including copies of all adapted/modified surveys are available on the project’s OSF page (https://osf.io/3bmqz/).

#### Measures

##### Student Survey

***Statistics Anxiety.*** Statistics anxiety was measured by the Statistics Anxiety Rating Scale (STARS; Cruise et al., 1985). The three anxiety subscales (Hanna et al., 2008; Papousek et al., 2012) of the STARS were used (23 items in total): test and class anxiety (8 items), interpretation anxiety (11 items), and fear of asking for help (4 items). Each item describes a situation involving statistics such as “Doing an examination in a statistics course” (test and class anxiety), “Interpreting the meaning of a table in a journal article” (interpretation anxiety), or “Going to ask my statistics teacher for individual help with material I am having difficulty understanding” (fear of asking for help). Participants were asked to indicate how much anxiety they feel in those situations on a Likert scale ranging from 1 = “no anxiety” to 5 = “a great deal of anxiety”.^12^

Several items use outdated language and were modified to reflect modern equivalents (e.g., “Asking one of my teachers for help in understanding a printout” was changed to “Asking one of my teachers for help in understanding statistical output”). These modifications are the same as those made in Terry et al. (2023).

An attention check was also included in this scale, which asked participants to “Please select ‘1 – no anxiety’ for this question”.

***Mathematics Anxiety.*** Mathematics anxiety was measured with the Revised Mathematics Anxiety Rating Scale (R-MARS; Baloğlu & Zelhart, 2007). There are three subscales in the R-MARS which measure mathematics test anxiety (15 items), numerical task anxiety (5 original items plus 4 modified items – see the Modified STARS and R-MARS section below for modification details), and mathematics course anxiety (5 items). Each item describes a situation involving mathematics such as “Taking an exam in a math course” (mathematics test anxiety), “Being given a set of division problems to solve” (numerical task anxiety), or “Listening to another student explain a math formula” (mathematics course anxiety). Participants were asked to indicate how much anxiety they feel in those situations on a Likert-type scale ranging from 1 = “no anxiety” to 5 = “a great deal of anxiety”.

Where the local context required it, items were modified to reflect local equivalents of US terms (e.g., in the UK, “Taking the *math* section of a *college* entrance exam” was changed to “Taking the *maths* section of a *university* entrance exam”).

***Modified STARS and R-MARS***. The modified versions of the STARS (STARS-M) and R-MARS (R-MARS-S) used in Terry et al. (2023) were also included. In these versions, the original STARS items were revised to reflect mathematics-related situations (e.g., “Doing the coursework for a statistics course” was changed to “Doing the coursework for a mathematics course”) and the original R-MARS statements were revised to reflect statistics-related situations (e.g., “Walking into a mathematics class” was changed to “Walking into a statistics class”). The response scales were kept the same as the originals.

Three items in the original STARS were not easily distinguishable as being about either mathematics or statistics so equivalent items were not created (“Arranging to have a body of data put into the computer”, “Reading an advertisement for a car which includes figures on miles per gallon, depreciation, etc.”, and “Trying to understand the odds in a lottery”). Additionally, one item on the original R-MARS was deemed untranslatable to a statistics context so, again, an equivalent was not created (“Reading a cash register receipt after your purchase”). These items are, therefore, identical to the original scales. When creating composites of the STARS-M and the R-MARS-S, the original items should be included instead so that both modified scales have the same number of items as their originals (23 for the STARS-M and 20 for the R-MARS-S).

The exploratory factor analysis in Terry et al. (2023) indicated that the R-MARS numerical task anxiety subscale was the only subscale where the revised items did not load onto the same factor as the corresponding original items. We believe the inconsistency in factor loadings in the original study could be because the modifications were not equivalent. For example, “Being given a set of numerical problems involving addition to solve on paper” was modified for the statistical context to “Calculating the sum of squared deviances by adding the squared deviances together” and, although the two both involved addition, the latter would be less familiar to participants and, thus, could be perceived as more a complex mathematical task. To rule out the possibility that the original and modified items loaded onto separate factors due to differences in perceived complexity, we re-modified the numerical task anxiety items and added these to the present version (as well as the original modifications, for comparison). Four items (R-MARS-S-NUM) were modified from mathematics items to statistics items whilst keeping the language more consistent (e.g., “Being given a set of numerical problems involving addition to solve on paper” was modified to “Being given a set of statistical problems involving addition to solve on paper”) and four items (R-MARS-NUM) were changed from our original modifications back to mathematics but matching the more complex language used (e.g., “Calculating the sum of squared deviances by adding the squared deviances together” has been modified to “Finding the codomain of the function h(x, y) = x + y when x = {3,4,5,6} and y = {5,7,9,13}”).

An attention check was also included in the STARS-M, which asked participants to “Please select ‘5 – a great deal of anxiety’ for this question”.

***Trait Anxiety.*** Trait anxiety was measured using the trait subscale of the State Trait Inventory for Cognitive and Somatic Anxiety (STICSA; Ree et al., 2008). The STICSA has been developed and evidenced to differentiate anxiety from depression more effectively than other popular anxiety measures (e.g., the State-Trait Anxiety Inventory (STAI); Spielberger, 1983; Tindall et al., 2021). The trait subscale is further broken down into cognitive (10 items) and somatic symptoms (11 items). Cognitive symptoms are measured with statements such as “I cannot concentrate without irrelevant thoughts intruding” and somatic symptoms are measured with statements such as “My heart beats fast”. Participants are asked to indicate the extent to which each item is true of them on a Likert scale ranging from 1 = “not at all” to 4 = “very much so”.

An attention check was also included in this scale, which asked participants to “Please select ‘1 – not at all’ for this question”.

***Test Anxiety.*** Test anxiety was measured with the Revised Test Anxiety Scale (R TAS; (Benson & El-Zahhar, 1994). The scale contains four subscales: 7 worry items (e.g., “During tests I find myself thinking about the consequences of failing”), 6 tension items (e.g., “During tests I feel very tense”), 5 test-irrelevant thinking items (e.g., “During tests I find I am distracted by thoughts of upcoming events”), and 7 bodily symptoms items (e.g., “I get a headache during an important test”). We included 5 items later removed by Benson and El-Zahhar (1994) to form a 20-item scale, which secondary researchers may also wish to remove (see Benson and El-Zahhar, 1994, for details). Participants were asked to respond to each item in terms of how they feel when taking tests in general on a scale of 1 = “almost never” to 4 = “almost always”.

***Fear of Negative Evaluation.*** Following recommendations by Carleton et al. (2011), fear of negative evaluation was measured using the Brief Fear of Negative Evaluation Scale – Straightforward (BNFE-S; Leary, 1983; Rodebaugh et al., 2004). The scale contains 8 items, including statements such as, “I am afraid that people will find fault with me” and “I often worry that I will say or do the wrong things”. The BNFE-S omits the reverse-scored items in the original BNFE scale (items 2, 4, 7, and 10), which were found to be measuring a different construct (Carleton et al., 2011). Participants were asked to indicate how characteristic each item is of them on a Likert scale ranging from 1 = “not at all characteristic of me” to 5 = “extremely characteristic of me”.

An attention check was also included in this scale, which asked participants to “Please select ‘3 – moderately characteristic of me’ for this question”.

***Social Interaction Anxiety and Performance Anxiety.*** Social interaction anxiety and performance anxiety were measured using the experienced fear/anxiety dimension of the Liebowitz Social Anxiety Scale – Self Report (LSAS-SR; Baker et al., 2002; Liebowitz, 1987). The scale is broken down into social interaction anxiety (12 items, e.g., “Talking with people you don’t know very well”) and performance anxiety (12 items, e.g., “Participating in small groups”). Participants were asked to indicate how anxious they would feel in each situation on a Likert scale ranging from 0 = “not at all” to 3 = “very much so”.

Some LSAS-SR items were adapted to respect local laws/norms in Saudi Arabia. Specifically, “Drinking with others” was reworded to “Drinking coffee with others”, “Urinating in a public bathroom” was changed to “Using a public bathroom”, and “Trying to make someone’s acquaintance for the purpose of a romantic/sexual relationship” was changed to “Making someone’s acquaintance for the purpose of making a marriage proposal”.

***Intolerance of Uncertainty.*** Intolerance of uncertainty was measured using the Intolerance of Uncertainty Scale – Short Form (IUS-SF; Carleton et al., 2007). The scale contains 2 subscales, Prospective Anxiety and Inhibitory Anxiety, each with 6 items. The Prospective Anxiety subscale includes statements such as, “The smallest doubt can stop me from acting”. The Inhibitory Anxiety subscale includes statements such as, “It frustrates me not having all the information I need”. Participants were asked to indicate how characteristic each item is of them on a Likert scale ranging from 1 = “not at all characteristic of me” to 5 = “entirely characteristic of me”.

***Creativity/Non-Creativity Anxiety.*** Creativity/Non-Creativity Anxiety was measured using the Creativity Anxiety Scale (Daker et al., 2020). The scale contains 16 items: 8 creativity anxiety items (e.g., “Having to solve a problem for which the solution is open-ended”) paired with 8 non-creativity items (e.g., “Working in a situation where there is an established correct and incorrect way of doing things”). Participants were asked to indicate how much each situation would make them feel anxious on a Likert scale ranging from 0 = “not at all” to 4 = “very much”.

An attention check was also included in this scale, which asked participants to “Please select ‘2 – a little’ for this question”.

***Analytic Thinking.*** Analytic thinking was measured using a revised version of the Cognitive Reflection Test (CRT; Frederick, 2005), developed by Shenhav et al. (2012). We selected a revised version because participants were less likely to be familiar with it than the original. Like the original, the revised CRT contains three word-problems, each of which requires a numerical response. Questions are open-ended, but respondents typically give either the correct response (indicating greatest analytic thinking), a single incorrect and intuitively compelling response, or varying incorrect and unintuitive responses. The data set contains the raw numerical responses given by participants so that researchers can code them according to their chosen criteria.

Participants were also asked, “You have just answered three reasoning problems. How many of them do you think you answered correctly?” and – to help ensure the integrity of the revised CRT – “You have just answered three reasoning problems. Did you look any of the answers up online?”, to which they could respond “Yes” or “No”.

***Belief in God/s.*** Participants’ belief in God/s was recorded using a single item. Participants were asked, “How strongly do you believe in God (or gods) from 0–100? If you are certain that God (or gods) does not exist, then enter “0” and if you are certain that God (or gods) does exist then enter “100”.” Possible responses ranged between 0 and 100.

***Self-Efficacy.*** Self-efficacy was measured with the 8-item New General Self Efficacy Scale (NGSE; Chen et al., 2001), which contains items such as “When facing difficult tasks, I am certain that I will accomplish them”. Participants were asked to indicate the extent to which they agree with each statement on a Likert scale of 1 = “strongly disagree” to 5 = “strongly agree”.

An attention check was also included in this scale which asked participants to “Please select ‘4 – agree’ for this question”.

***Persistence.*** Persistence was measured with the persistence subscale of the Attitude Towards Mathematics Survey (ATMS; Miller et al., 1996), which contains 8 items such as “If I have trouble understanding a problem, I go over it again until I understand it”. Although the ATMS as a whole focusses on mathematics, the persistence subscale items refer to academic persistence more generally. Some items were modified to make them more appropriate for the higher education context. Specifically, in item 3 the words “in the book” were removed, in item 6 the words “hope that the teacher explains it” were changed to “hope that it is explained”, and the word “homework” was removed from items 2, 7, and 8. Item 4, “If I have trouble solving a homework problem in the book, I copy down the answer in the back of the book if it is available”, was removed because the required modifications would have changed the meaning too far from the original. All items except 1 and 7 are reverse scored. Participants were asked to indicate the extent to which they agree with each statement on a Likert scale of 1 = “strongly disagree” to 5 = “strongly agree”.

An attention check was also included in this scale, which asked participants to “Please select ‘4 – agree’ for this question”.

***Mathematics Education.*** Participants were asked for their highest level of pre-university mathematics education (GCSE or A Level or international equivalents), the grade they received at each level, and how long ago (in months) they took each qualification. These questions were modified for the local context of each partner university and, consequently, some include additional questions (see codebook for full details). Note that grades are in their raw form and will need to be standardised before they can be compared.

***Statistics Grades (Self-Reported).*** We asked participants whether they had previously taken any university-level statistics modules and, for those that had, to self-report their grades for these modules. Grades are in their raw form, but we also provide grading scale/system information for each university to enable standardisation (see Grade Data, below).

***Degree Course Details.*** Participants were asked to indicate their university, (intended) major subject of study (i.e., the subject of the degree that they are pursuing), their current year of study, and whether they were studying any other (i.e., non-statistics) mathematics-based modules on their degree. Where the local researchers already knew these details (e.g., they were only sharing the survey with their own students) these questions were omitted to reduce the length of the survey and the information was instead added into the data during data processing.

***Demographics.*** Participants were also invited to provide their age (in years), gender identity, ethnicity, and whether they have been diagnosed with a specific learning difference (SpLD), such as dyslexia or dyscalculia.

***Attention Check.*** In addition to the attention checks embedded within the measurement scales, participants were presented with the following at the end of the survey: “Please indicate whether you feel you have answered the previous questions carefully and truthfully. Answering ‘yes’ will ensure that your data is included in our analyses. Answering ‘no’ will mean that your data is excluded from our analyses but will have no impact upon you (i.e., you will still earn your incentive for taking part)”. Participants could respond “Yes, I have answered all questions carefully and truthfully” or “No, I have not answered the questions carefully and truthfully”.

***Survey Metadata.*** The dataset also contains selected metadata that was automatically collected by Qualtrics (Qualtrics, Provo, UT), which researchers may find useful. Specifically, we include the percentage of the survey completed, the time it took to complete the survey, and the dates participants began and finished the survey.

***Identifiers.*** We have also added relevant identifiers. Specifically, the country in which the survey was taken, the language in which the survey was taken, the survey ID (because some surveys were made available to students in more than one university), and a randomly generated participant ID, which replaced the participant-generated ID code for anonymisation purposes. The type of incentive offered to students and the context (inside or outside of class) in which the survey was completed has also been recorded.

##### Instructor Survey

***Statistics Module Details.*** The instructor survey asked for the following information about each module: Name and/or code, start and end dates, the statistical software taught, the approximate content of the modules (via a checklist of different statistics topics), the primary academic discipline of the instructors, the mode(s) of teaching and number of hours per format (e.g., 1-hour online lecture, 2-hour in-person workshop), the types, format, and date of assessments, how assessments were graded, opportunities for formative feedback, average grade from previous cohorts, and any other information that would be useful to contextualise the assessment information.

##### Grade Data

***Statistics Grades (Official).*** At universities where it was approved by the local ethics committees, we asked student participants to provide their names and/or student ID codes, so that the grades for the statistics modules they were taking at the time they completed the survey could be obtained from their university records. Note that grades are in their raw form, but we also provide grading scale/system information to enable standardisation.

#### Procedure

##### Student Survey

Upon receiving the invitation to take part, students were directed to the online survey where they read the information sheet and provided consent before continuing. Participants were then asked to complete an eligibility check (if they had not been pre-screened), and to provide their name and/or student ID code (to obtain grade data from student records, where relevant), a unique participant ID code (to withdraw their data, if desired), and their primary degree subject and statistics module names (if researchers were unsure of these details in advance). All participants then completed the first block of measurement scales containing all four measures of statistics anxiety and mathematics anxiety, randomised at the measure and item level. This block was presented first because it contained the measures most critical to the primary study and if participants did not proceed to the next block, their data would still be useful. The second block of measurement scales – also randomised at the question and item level – contained measures of trait anxiety, test anxiety, fear of negative evaluation, social interaction/performance anxiety, creativity anxiety, intolerance of uncertainty, self-efficacy, persistence, and the revised cognitive reflection test (CRT). The question asking about participants’ belief in God/s was randomly presented before or after the revised CRT. The two follow-up questions about the revised CRT were then asked. Participants were next asked about their pre-university mathematics education, their statistics grades from previous modules at university (if applicable), the year of their degree course, and demographics. Finally, participants answered the final attention check question, were debriefed, and if required, redirected to collect their survey incentives. The median completion time for the survey was 30 minutes.

##### Instructor Survey

Upon receiving the invitation to take part, statistics module instructors were directed to the online survey where they read the information sheet and provided consent before continuing. Participants were first reminded that they should complete the instructor survey once for every statistics module that the student participants were taking at the time of completing the student survey and provided with a unique code they could use if they later wished to remove their data. The survey then requested (in order) the university name, the statistics module name and/or code, and the start and end dates of the module. Participants could then select the software(s) taught on the module, whether the module was frequentist, Bayesian, both, or other, and select the topics taught from a checklist (e.g., ANOVA, Bayes factors, Data visualisation). We then asked whether the module was taught by the mathematics/statistics department or from the students’ main discipline (e.g., psychology faculty that teach statistics). The survey then requested the percentage of in-person teaching and whether there was less than usual due to COVID-19. We then asked for details about the mode of teaching (e.g., lectures, practicals), including how many hours per week were spent on each, whether they were online or in-person, and synchronous or asynchronous.

The next section was about module assessments. We asked for the type of assessment (e.g., exams, coursework), the percentage of the final grade each type contributed to, the length of any timed assessments, whether assessments were online/in-person (where appropriate), the date of exams/deadlines for coursework, and the scale used for grading (e.g., numeric continuous, letter grades). Where respondents reported using regular testing, we also asked for the frequency and format (e.g., quizzes, tasks) of testing, whether they were timed, and whether all grades counted towards the final, overall grade. Next, instructors could indicate the types of any formative assessment (e.g., verbal/written, peer/instructor), what the average final overall grade for the module usually is, and, finally, instructors were invited to record any additional information about their assessments that could be useful for contextualising their data.

##### Grade Data

Where permitted by collaborating universities’ ethics committees and legal teams, grade data was obtained by the collaborating researchers and shared with the lead researcher using password-protected files.

### 2.6 Quality Control

#### Attention Checks

At the end of the student survey, participants were asked whether they had answered all questions truthfully and carefully, to which 10,281 (81.8%) responded “yes”, 172 (1.4%) responded “no”, and 2,117 (16.8%) responses are missing (where participants did not reach that stage of the survey).

Additionally, six attention checks were embedded within the measurement scales which asked participants to select a specific response (e.g., “please select ‘1 – strongly disagree’”). There were two in the first block which contained the statistics and mathematics anxiety measures and four in the second block which contained all other scales. In the first block, 2,052 (16.3%) of participants responded incorrectly to the first check and 2,194 (17.5%) responded incorrectly to the second. In the second block, the number of students responding incorrectly to each check were 2,779 (22.1%) to the third, 2,812 (22.4%) to the fourth, 2,872 (22.9%) to the fifth, and 2,752 (21.9%) to the sixth.

#### CRT Check

To help ensure the integrity of the revised CRT, participants were asked “You have just answered three reasoning problems. Did you look any of the answers up online?”, to which they could respond “Yes” or “No”. There were 382 (3.0%) participants that answered “Yes’ to this question.

### 2.7 Data anonymisation and ethical issues

#### Ethics

This study was approved (ER/JLT26/7) by the Sciences & Technology Cross-Schools Research Ethics Committee (C-REC) at the University of Sussex in adherence to the British Psychological Society’s Code of Human Research Ethics (2018). Partner universities were covered by the overarching University of Sussex ethics approval, but were asked to check with their own ethics boards whether further approval was required at the local level and, if necessary, to obtain it before beginning data collection. Ethics approval documentation is available here: https://osf.io/2aumd/.

For those universities that shared students’ grade data with us, a data protection agreement was in place to allow the legal transfer of the non-anonymised data (i.e., student names and/or ID codes) required to obtain, share, and link grades to participants’ survey data.

#### Anonymisation

Raw data have and will only ever be available to the research leads at the University of Sussex. To anonymise the data for sharing, the student names and ID codes have been replaced with a randomly generated unique ID code, and the demographic variables and some course/module details from the student surveys have been edited as required to ensure that participants are not identifiable via a combination of these data. Specifically, students’ age, degree major, and any specified non-statistics mathematics modules have been categorised, gender identities and SpLDs have been partially re-categorised, and ethnicity data have been removed completely. Full details on how the data have been processed for anonymisation is available in the codebook and data processing notes (available here: https://osf.io/374vn/).

### 2.8 Existing use of data

At the time of writing, there are no published articles or other outputs originating from this data. However, following the embargo period, researchers will be able to register their planned secondary analyses on an open document, which we encourage use of to prevent duplication of efforts.

## (3) Dataset description and access

### 3.1 Repository location

DOI 10.17605/OSF.IO/MHG94

### 3.2 Object/file name

The dataset is available in its complete form (i.e., the combined and matched student survey, instructor survey, and grades) and – due to its size – also in its component parts, which can be rematched using the `unique_id` variable. Accompanying the data is a detailed codebook. The files are named as follows:

- SMARVUS_complete.csv – all data
- SMARVUS_demo_meta.csv – id, demographics, Qualtrics meta-data, and key identifiers
- SMARVUS_measures.csv – id, measurement scales
- SMARVUS_maths_edu.csv – id, prior mathematics education data
- SMARVUS_stats_edu.csv – id, statistics education data (from official records and self-reported)
- SMARVUS_codebook.csv – the codebook

### 3.3 Data type

Partially processed primary data.^13^

### 3.4 Format names and versions

All versions of the dataset are available as .csv files, which can be opened using most spreadsheet and statistics software.

### 3.5 Language

All data are stored in English (UK), except proper nouns (e.g., names of pre-university mathematics qualifications). Free-text responses were mostly short and straightforward to translate (e.g., degree major or gender identity) so were translated back into English by the lead author using Google Translate. Where a translation was ambiguous, it was clarified with native speakers.

### 3.6 License

The data and supplementary materials are licenced under a CC BY 4.0 licence.

### 3.7 Limits to sharing

Data will be under embargo until 1st of October, 2024 to allow the authors sufficient time to publish from it first. During this time, data will be made available upon request, provided the intended research does not overlap with projects being undertaken by the present authors.

### 3.8 Publication date

1st of October, 2024

### 3.9 FAIR data/Codebook

Data are stored in .csv format on the OSF, along with a detailed codebook and all materials, using a CC BY 4.0 licence.

## (4) Reuse potential

The SMARVUS dataset has the potential to address many important questions, particularly regarding statistics and mathematics education, anxiety, psychometrics, and survey methodology. It uniquely facilitates cross-lingual and cross-cultural comparisons and the larger-than-usual sample size is more likely to produce reliable and robust estimates. Below, we highlight just some of the ways this could benefit specific fields.

### Statistics Education

These data enable the exploration of relationships between many constructs. For example, a much-debated question is whether statistics anxiety affects achievement (e.g., statistics module grades). A recent meta-analysis of this relationship produced a non-significant effect size of just *Z* = |0.07| (Trassi et al., 2022). However, the authors noted considerable variability in their systematic review, explaining it may be attributed to moderators, such as pre-university mathematics grades and self-efficacy. Another review identified mode of assessment as a potential moderator (Terry & Field, 2023). These moderators could be tested with the SMARVUS dataset.

Variability could also be due to the multi-dimensionality of the STARS (Cruise et al., 1985). There are three subscales that measure statistics attitudes, not statistics anxiety (Hanna et al., 2008; Papousek et al., 2012), thus should not be conflated. The data required for Trassi et al. (2022) to separate these subscales were unavailable, forcing them to use composite scores. A large-scale analysis of the relationship between statistics anxiety and achievement using the anxiety subscales alone is possible with the SMARVUS dataset.

Trassi et al. (2022) further note that studies in their meta-analysis mainly tested psychology students within Europe and North America and many had low sample sizes, which the SMARVUS dataset addresses. Such limitations are pervasive in psychology (Ioannidis, 2005; Rad et al., 2018), so these data could benefit many other research questions in the same ways. Furthermore, the sample is sufficiently large to enable multi-level modelling to estimate variation across different languages, geographic regions, or educational systems.

### Construct Validity

To understand how generalisable research is, the scales we use to measure constructs must be validated in different populations (Flake, 2021). This includes ensuring adaptations (e.g., translations) are valid and reliable, and that different groups respond to measures in the same ways, such that the factor structure, loadings, and item intercepts are equivalent (i.e., are *measurement invariant*).

Our student survey included eight scales adapted to 21 languages. We also modified some scales to be appropriate for the local context. In most cases, this was minimal (e.g., changing “college” to “university”). However, we made more substantial modifications to our measure of social interaction and performance anxiety – the LSAS-SR (Baker et al., 2002) – for use in Saudi Arabia (e.g., modifying inappropriate references to alcohol and dating). Validating adapted scales would ensure these versions are appropriate for use in different countries and cultural contexts, opening up fresh opportunities for cross-cultural research.

Our data could also be used for measurement invariance testing. There is a dearth of invariance testing for most psychological scales (D’Urso et al., 2022), so there are many gaps to be filled. For example, we know that mathematics anxiety scores vary between cultures (Hunt et al., 2021), which could be indicative of cultural non-invariance. If that is the case, the generalisability of predominantly Western research findings should not be assumed. Such findings might be misleading for other cultures, with consequences for education. This dataset could address this problem via the cross-cultural investigation of mathematics anxiety scale properties.

### Cognitive Reflection Test (CRT)

The data includes responses to a revised version of the Cognitive Reflection Test (CRT; Shenhav et al., 2012), a hugely popular measure of reflective thinking tendencies, the original (Frederick, 2005), having been cited over 6000 times, according to Google Scholar. Projects are underway to test the psychometric properties of Shenhav et al.’s (2012) version and, assuming the scale shares key properties of the original (e.g., excellent validity, reasonable reliability, and incorrect responses converging on the same typical response), the SMARVUS opens up opportunities for research into cross-cultural and gender comparisons of cognitive reflection and its relationship to various types of anxiety.

### Survey Methodology

For survey-based research to be robust, it is essential that care and attention are employed by respondents. One study found 10–12% of responses to long surveys by undergraduates completing it for course credit are given without such care (Meade & Craig, 2012). Some researchers have proposed using attention checks to help identify and eliminate such responses (Huang et al., 2012). The present study included attention checks within the survey measures, asking participants to choose a particular response option, and an ‘amnesty’ at the end, asking if they had answered carefully and truthfully throughout. SMARVUS data could be used to compare the effectiveness of these checks with other measures of careless responding, such as response time and ‘long-string analysis’ (providing the same response to all items on a scale; Curran, 2016).

### Pedagogy

Finally, we suggest the SMARVUS dataset has unique pedagogical reuse potential. First, students might find a dataset related to mathematics and statistics anxieties to be relatable, something qualitative evidence suggests can aid learning (e.g., Blackburn, 2015) and reduce anxiety (e.g., Trakulphadetkrai, 2017) in statistics education. Second, using these data in a statistics class would give instructors an opportunity to make students conscious of any anxieties, show them they are far from alone, and encourage students to notice and, subsequently, challenge the influence anxiety may be having on their attitudes and behaviours regarding learning statistics. Third, there are general benefits of using authentic, secondary data in statistics education that could further enhance the specific benefits. For example, students can learn data processing strategies that are usually unavailable with pre-prepared datasets – such as dealing with missing data – alongside statistical procedures and tests. Additionally, students who use this data for research projects (e.g., undergraduate dissertations) could do so without needing to apply for ethics approval or worrying about recruiting a large enough sample, and could present their work at conferences and in publications, as previously done by Long & Chalk (2020) with Grahe et al.’s (2018) *Emerging Adulthood Measured at Multiple Institutions 2* (EAMMi2) data.

## Footnotes

We use the term ‘module’ to describe the smaller units that make up a degree course (often lasting one semester) to distinguish them from full degree programmes, which we refer to as ‘courses’.

We also added a single item measure of participants’ belief in God/s to test a CRT-related research question, outside of our core aims.

The period of data collection coincided with the COVID-19 pandemic, which affected teaching delivery (e.g., the move to online learning, some details of which were captured by the instructor survey) and general anxiety levels may have been higher than usual.

Here, the UK includes the devolved nations of England, Scotland, and Northern Ireland (we did not collect data in Wales). Note, however, that the devolved nations have different education systems both pre-university (e.g., different mathematics exams) and during university (e.g., different degree durations).

16.7% of collaborating researchers did not provide this data (typically, because they were unable to).

A limitation of these modes of recruitment is that it is usually not possible to determine how many students were exposed to the invitation to take part. Consequently, we do not provide participation rates as the data would be incomplete and/or inaccurate.

We did not collect data on which device was used, but note that the device used could be a source of heterogeneity.

Although we recognise that “female” and “male” refer to biological sex and that “woman” and “man” refer to gender identity, the adapted response options to the question asking participants’ gender identity were inconsistent with some listing “woman” and “man”, others listing “female” and “male”, and others listing “woman/female” and “man/male”, so we have merged the responses.

An anonymised list of the ways participants described their gender is available in the OSF supplementary materials.

See the supplementary materials for the frequency of degree major categories below 1%.

Some universities also provided students’ grades from their previous modules. This data will be provided as a supplementary file on the OSF at a later date.

We do not provide reliability coefficients for this or any of the other measures because such coefficients should be calculated for the specific subsample chosen for any secondary research studies.

Our aim was to provide data as close to its raw form as possible, after translations and anonymisation. Whether and how each variable has been processed is detailed in the ‘Data Processing Notes’ column of the code book and is summarised here: https://osf.io/6n9ye.
