# Supplementary material for: Data from an International Multi-Centre Study of Statistics and Mathematics Anxieties and Related Variables in University Students (the SMARVUS Dataset)
Source: J Open Psychol Data. 2023 May 29;11:8. doi: 10.5334/jopd.80 (PMC12270023; doi:10.5334/jopd.80)
Supplement: PR File 1. — Peer Review History. [file jopd-11-80-pr1.zip › pr1-jopd-80_terry/peer_review_comments.docx]

Reviewer A:

The paper describes a large dataset including data on anxieties (especially those that are related to statistics and mathematics) in university students. The data were collected at universities in different countries all over the world. In addition to different aspects of anxieties self-efficacy, persistence, the cognitive reflection test and demographic variables were assessed.

Due to the large number of constructs covered and the variety of countries included the dataset offers many opportunities to reuse the data.

I only have a few comments that the author(s) could consider in a revision of the manuscript.

In the first section (‘Background’) the focus is put on the question whether mathematics anxiety and statistics anxiety are independent constructs or not. Although I understand that the clarification of this question was the main reason for the data collection, this question (‘jangle fallacy’) appears quite specific. Therefore, I would recommend to frame this section in a somewhat broader way. For example, the relevance of mathematics /statistics anxiety in the university context could be addressed or relationships to related constructs could be described in more detail.

When reading the methods section I got the impression that some information is redundant. For instance, information on the student survey, the instructor survey and students’ grade data is given in the sections 2.1, 2.4. and 2.5. The authors could check this and shorten overlapping text parts or restructure this section.

If space is an issue the author(s) might consider omitting Figure 1, since the essential information is already contained in Table 1.

Is it possible to match the data of the students to the corresponding courses / instructor surveys?

Was the participation in the student survey /instructor survey voluntary? Is there any information about participation rates or selectivity of participation? And is there any information on the effects of incentives on participation rates?

------------------------------------------------------

Reviewer B:

General description:

The authors provide the international data on anxieties (e.g., mathematics anxiety and statistics anxiety) in an educational context. The data set includes also some personality traits, cognitive tasks, sociodemographic data, as well as achievement scores and information on statistical modules. The data set can be used to study the relationship between anxieties and correlates, to conduct international comparisons, and to test the psychometric properties of the measures.

General comment:

The manuscript is well-written. Most of the information was provided and described thoroughly. However, there are still some missing pieces and inconsistencies which should be addressed to improve the quality of the manuscript and data set.

Major points:

1) The objectivity of data collection is important in the type of studies that perform multi-center data collection. When collecting data, it is important to keep the conditions as equal as possible so that it does not negatively affect the results on the analyses. For example,

a) Context of data collection: Some data were collected online and some were collected as part of an in-class exercise. These contextual differences in data collection could bias the results.

b) The device used for data collection: The type of device (smartphone or PC) could also affect data quality. On the one hand, the screen of a smartphone is small, on the other hand, participants often answer an online questionnaire with a smartphone while on the road, therefore in a quite different context than when answering a questionnaire on a PC.

To ensure objectivity, it will be helpful if the authors could add this type of variables to the data set, if they were also collected.

2) The data set does not specify different types of missing values. Thus, in the data set, all missing values are indicated as “NA”, regardless of how they originated. For example:

a) Variable age: Some students did not provide plausible values and some students did not answer the question. These two types of missing values should be identifiable in the data set: missing values due to "implausible values" and missing values due to "non-response". Implausible values might be of interest for researchers studying careless responding, for example.

b) Another type of missing values arises on items that were removed from the questionnaire due to the requirements of local ethics authorities, or on items that were inappropriate in the local context, or because additional items were included for some local contexts/universities (e.g., related to mathematics education). This type of missing values should be defined as "not administered" and separated from missing values due to non-response.

It will be helpful if the authors define different types of missing values in the data set.

3) On the OSF repository, student survey questionnaires in local contexts/universities are provided. It will be easier for users to find a questionnaire administrated in a particular context/university if the authors rename the files. For example, if the file name would contain the code of the country and the name of the university. Then the files would be listed in alphabetical order.

4) A check of the instruments described in Measure section and in the codebook revealed a number of inconsistencies. For example:

a) Regarding the stat/math anxiety scales in the Measures section, it is difficult to understand why the number of items in the codebook and in the Measures section do not match. According to the description of the R-MARS in the Measures section, the RMARS contains 20 items. However, the codebook actually indicates that 24 items were actually collected (R-MARS plus additional R-MARS NUM items). It will be nice if the authors mention already in the description of the RMARS that four additional items were added to the scale, with a reference to the corresponding text passage in the paper where it is described in detail. Otherwise, it is confusing.

b) Regarding the STARS-M, it will be nice if the authors explicitly note that three items are identical to the STARS, so that the two scales contain 23 items. This also applies to the R-MARS-M and R-MARS.

c) Regarding the test anxiety scale, according to the codebook, the scale contains 25 items; in the Measure section, only 20 items (summed over the subscales) are reported. The subscale tension is not mentioned. Also, the number of items in the subscales does not match the infomation in the codebook. It will be nice if the authors correct it.

5) For the instructor survey, it is not clear whether all instructors were surveyed in English or whether the instructor survey questionnaires were also translated into native languages. The OSF repository contains only the English version of the instructor survey. It will be helpful if the authors could add this information in paper in the section Materials/Survey Instruments.

6) In the OSF repository, in the folder Ethics, the files are available for some local contexts/universities that are not included in Table 1. For example, Ankara Science University, IDC Herzliya, Ryerson (University). It will be nice if the authors could check it and eliminate the inconsistencies.

7) It will be helpful if the authors check the date variables in the data set. For example, the variables "start_date" and "end_date" (the 8th and 9th variables in the data set) contain values that do not provide a date/time (e.g., first line: unique_id= d7a4bc67; start_date =44324; end_date=44324).

Minor points:

1) According to the journal guidelines, keywords should be separated by a semicolon (not comma).

2) It would be nice if the numbers greater than 1,000 are written consistently (e.g., in Table 1, Egypt N=1,390 (not N=1390)).

3) In the sentence "The mean completion time for the survey was 30 minutes).", a parenthesis is superfluous.
