# Supplementary material for: Data from an International Multi-Centre Study of Statistics and Mathematics Anxieties and Related Variables in University Students (the SMARVUS Dataset)
Source: J Open Psychol Data. 2023 May 29;11:8. doi: 10.5334/jopd.80 (PMC12270023; doi:10.5334/jopd.80)
Supplement: PR File 1. — Peer Review History. [file jopd-11-80-pr1.zip › pr1-jopd-80_terry/smarvus_peer_review_response_letter_.docx]

**Editor’s Comments**

***Editor Comment (EC) 1:*** First, Reviewer A asked for some clarifications regarding the consent procedure, incentives, nonresponse, and potential sample selection. I agree that it would benefit the manuscript if respective information were given or, if not available, discussed as potential limitations. Furthermore, Reviewer B asks for more information regarding the data collection procedure. It would be very helpful if respective information could be provided.

***Author Response to the Editor (ARE) 1:*** As Reviewer A requested, we have clarified that participation in both the student and instructor surveys was voluntary (see Section 2.4). We have also made it explicit in that section that the student samples were convenience samples.

However, we have not been able to provide information about participation rates, because only 7.8% of collaborators indicated they could provide exact figures. In most cases, this was due to the manner in which the study was advertised at their site. For example, in cases where SONA or social media was used to recruit students in exchange for course credits, we have no way of knowing how many students actually saw the advert. It would also be very difficult to obtain accurate summaries of the number of students eligible to take part at each site (regardless of whether they had seen the advert), as in many cases students were recruited from outside of the courses collaborators taught and the required information would be held by the University and not made available to collaborators due to data protection concerns. We have noted this omission as a limitation (see footnote 6 in Section 2.4). Accordingly, we do not have data on the effect of incentives on participation rates.

As Reviewer B requested, we have added a variable to the data & codebook (`participation_context`) that captures whether the data was collected as part of an in-class exercise or in students’ own time, outside of class. We have also added corresponding percentages to the manuscript (see Section 2.4). We do not have any data on the type of device used to respond to the surveys, so could not add that, but have added a footnote that explains we do not have data on which device was used and that this could be a source of heterogeneity (see footnote 7 in Section 2.4). We have also clarified that the instructor survey was only administered in English (see Section 2.5).

***EC 2:*** Second, Reviewer B made several suggestions to increase the richness of the data set (e.g., coding of missing values) and the organization of the files in the OSF. I recommend looking into whether you can improve on these points.

***ARE 2:*** The coding of missing values has been applied as suggested by Reviewer B and the data paper (see Section 2.5) and codebook (see Response Options for the Student Survey section) have been adapted accordingly. Specifically, we have re-coded the age variable so that implausible values are recoded from `NA` to `Implausible` and have also re-coded values that were not administered as opposed to just not having been responded to as `Not Administered` instead of `NA`.

With regard to the naming of the survey files, we agree with the reviewer's point about making the surveys easier to find. However, we have not done this by using the university names consistently, as the reviewer suggested, but instead tried to make it clearer how to find a given survey using the existing system on the OSF page, here: <https://osf.io/u58fp/?view_only=9d70d0facfeb476987f32d4ab156ecdc>). To elaborate, the files are named in accordance with the `survey_id`, which is the name used to identify each survey in Qualtrics and is a variable in the data. The `survey_id` is usually the unique part of the university name (e.g., `king_fasial`, `leeds_trinity`, `izmir_eco` etc.), but is sometimes a country name where one survey was used to collect data at multiple universities (e.g. `poland`, `hungary`) and there are also some cases where a university has two surveys because they required slightly different versions for different incentives (e.g. `brighton_a`, `brighton_b`). Renaming the files as suggested - with university names only - would not work in such cases, but we hope that we have made it easier to find the surveys with this adjustment instead.

***EC 3:*** Third, Reviewer B noted that the number of items in the codebook and the number of items in each scale does not match. This is an important issue. Please correct these inconsistencies and/or explain in the manuscript the rationale for the mismatch. Similarly, also check whether the files in the “ethics” folder match the locations given in Table 1. Please note, that the reviewer also noted some data irregularities (e.g., regarding the date).

***ARE 3:***  The mismatch in the Test Anxiety scale noted by Reviewer B has been corrected and we have also attended to the Reviewer’s other points regarding the description of measures, the naming of the ethics files, and the data irregularities. Specifically:

With regards to the extra R-MARS NUM items, we have made the suggested amendment. That is, we have mentioned the extra items in the Measures section and pointed to the next section where it is described in more detail. We have also made it clearer what the R-MARS NUM items are in the corresponding notes column of the codebook.

With regards to the STARS-M and R-MARS-S, we have added a sentence that makes it more explicit that several items are identical to the originals and the originals should be used in composite scores. We have also added notes for the relevant items in the codebook.

With regard to the inconsistencies between the codebook and the Measures section for the Test Anxiety scale, the number of items in the subscales has been corrected. Specifically, the codebook was correct and the error was in reporting a different version of the scale in the Measures section, so that has been edited. We could not, however, see where the tension scale was missing - it was and remains described in both the Measures section and in the codebook.

With regards to the organisation of files in the ethics folder, these have now been modified/removed accordingly. Two files did not match the locations given in Table 1 due to changes in the universities’ names since ethics approval was obtained and one was a university that withdrew after approval was granted.

With regards to the data irregularities, we had also spotted those since our original submission and these have now been fixed. The irregularities occurred due to the use of Excel reverting some of the dates back to date codes (the `44324` type numbers) and turned some of the ID codes into scientific notation. All data files can now be opened in Excel without this happening.

***EC 4:*** Fourth, I have noted that you provide the data and material under a “no derivative” license. This somewhat limits the reuse potential of the data. Please consider selecting a less restrictive license. Also, make sure to indicate whether the license only applies to the data or also to the instruments (and their translations). Do you have the right to distribute the scales under these conditions?

***ARE 4:*** Following further research, we agree that we can provide the data under a less restrictive licence and have instead applied a CC-BY license (see Section 3.6 and 3.9 and the licence section of the OSF page, here: <https://osf.io/m8gyr/?view_only=e791aaad78bb42259c01cd26aac3e44e>). We also make it clearer that this licence corresponds only to the data and supplementary materials (see Section 3.6).

***EC 5:*** Finally, in Section 3, please also describe the file containing the codebook. After all, the data is not usable without the codebook.

***ARE 5:*** The codebook has been described in Section 3.2, as suggested.

**Reviewer A Comments**

***Review A Comment (RAC) 1:*** In the first section (‘Background’) the focus is put on the question whether mathematics anxiety and statistics anxiety are independent constructs or not. Although I understand that the clarification of this question was the main reason for the data collection, this question (‘jangle fallacy’) appears quite specific. Therefore, I would recommend to frame this section in a somewhat broader way. For example, the relevance of mathematics /statistics anxiety in the university context could be addressed or relationships to related constructs could be described in more detail.

***Author Response to Reviewer A (ARRA) 1:*** We have not revised the background section as we believe it would be counter to the guidelines in the journal template which state, “This section should not constitute a full literature review of the topic area, but instead help highlight the literature that informed the motivation and purpose behind collection of the data and potential for use.”

***RAC 2:*** When reading the methods section I got the impression that some information is redundant. For instance, information on the student survey, the instructor survey and students’ grade data is given in the sections 2.1, 2.4. and 2.5. The authors could check this and shorten overlapping text parts or restructure this section.

***ARRA 2:*** The repetition is a side-effect of using the journal’s prescribed sub-headings which prevents us from restructuring. We believe that although there is a small amount of repetition, the main text covers different aspect of each mode of data collection.

***RAC 3:*** If space is an issue the author(s) might consider omitting Figure 1, since the essential information is already contained in Table 1.

***ARRA 3:*** Space is not an issue as far as we are aware and we believe that the additional regional summaries presented in Figure 1 are informative and worth keeping, so we have left this unchanged.

***RAC 4:*** Is it possible to match the data of the students to the corresponding courses / instructor surveys?

***ARRA 4:*** The data in `SMARVUS_complete.csv` is already matched across the student survey, instructor survey, and grade data. We have made that clearer in the document and specified that where we also provide subsets of the data, they can be re-matched using the unique id codes (see Section 3.2).

***RAC 5:*** Was the participation in the student survey /instructor survey voluntary? Is there any information about participation rates or selectivity of participation? And is there any information on the effects of incentives on participation rates?

***ARRA 5:*** See ARE 1, above.

**Reviewer B Comments**

***Review B Comment (RBC) 1:*** The objectivity of data collection is important in the type of studies that perform multi-center data collection. When collecting data, it is important to keep the conditions as equal as possible so that it does not negatively affect the results on the analyses. For example,

a) Context of data collection: Some data were collected online and some were collected as part of an in-class exercise. These contextual differences in data collection could bias the results.
b) The device used for data collection: The type of device (smartphone or PC) could also affect data quality. On the one hand, the screen of a smartphone is small, on the other hand, participants often answer an online questionnaire with a smartphone while on the road, therefore in a quite different context than when answering a questionnaire on a PC.

To ensure objectivity, it will be helpful if the authors could add this type of variables to the data set, if they were also collected.

***Author Response to Reviewer B (ARRB) 1:*** See ARE 1, above.

***RBC 2:*** The data set does not specify different types of missing values. Thus, in the data set, all missing values are indicated as “NA”, regardless of how they originated. For example:

a) Variable age: Some students did not provide plausible values and some students did not answer the question. These two types of missing values should be identifiable in the data set: missing values due to "implausible values" and missing values due to "non-response". Implausible values might be of interest for researchers studying careless responding, for example.
b) Another type of missing values arises on items that were removed from the questionnaire due to the requirements of local ethics authorities, or on items that were inappropriate in the local context, or because additional items were included for some local contexts/universities (e.g., related to mathematics education). This type of missing values should be defined as "not administered" and separated from missing values due to non-response.

It will be helpful if the authors define different types of missing values in the data set.

***ARRB 2:*** See ARE 2, above.

***RBC 3:*** On the OSF repository, student survey questionnaires in local contexts/universities are provided. It will be easier for users to find a questionnaire administrated in a particular context/university if the authors rename the files. For example, if the file name would contain the code of the country and the name of the university. Then the files would be listed in alphabetical order.

***ARRB 3:*** See ARE 2, above.

***RBC 4:*** A check of the instruments described in Measure section and in the codebook revealed a number of inconsistencies. For example:

a) Regarding the stat/math anxiety scales in the Measures section, it is difficult to understand why the number of items in the codebook and in the Measures section do not match. According to the description of the R-MARS in the Measures section, the RMARS contains 20 items. However, the codebook actually indicates that 24 items were actually collected (R-MARS plus additional R-MARS NUM items). It will be nice if the authors mention already in the description of the RMARS that four additional items were added to the scale, with a reference to the corresponding text passage in the paper where it is described in detail. Otherwise, it is confusing.
b) Regarding the STARS-M, it will be nice if the authors explicitly note that three items are identical to the STARS, so that the two scales contain 23 items. This also applies to the R-MARS-M and R-MARS.
c) Regarding the test anxiety scale, according to the codebook, the scale contains 25 items; in the Measure section, only 20 items (summed over the subscales) are reported. The subscale tension is not mentioned. Also, the number of items in the subscales does not match the infomation in the codebook. It will be nice if the authors correct it.

***ARRB 4:*** See ARE 3, above

***RBC 5:*** For the instructor survey, it is not clear whether all instructors were surveyed in English or whether the instructor survey questionnaires were also translated into native languages. The OSF repository contains only the English version of the instructor survey. It will be helpful if the authors could add this information in paper in the section Materials/Survey Instruments.

***ARRB 5:*** See ARE 1, above.

***RBC 6:*** In the OSF repository, in the folder Ethics, the files are available for some local contexts/universities that are not included in Table 1. For example, Ankara Science University, IDC Herzliya, Ryerson (University). It will be nice if the authors could check it and eliminate the inconsistencies.

***ARRB 6:*** See ARE 3, above.

***RBC 7:*** It will be helpful if the authors check the date variables in the data set. For example, the variables "start_date" and "end_date" (the 8th and 9th variables in the data set) contain values that do not provide a date/time (e.g., first line: unique_id= d7a4bc67; start_date =44324; end_date=44324).

***ARRB 7:*** See ARE 3, above.

***RBC 8:*** According to the journal guidelines, keywords should be separated by a semicolon (not comma).

***ARRB 8:*** The keywords are now separated by semicolons, not commas.

***RBC 9:*** It would be nice if the numbers greater than 1,000 are written consistently (e.g., in Table 1, Egypt N=1,390 (not N=1390)).

***ARRB 9:*** We have adjusted two values in Table 1 and one in section 2.6 to include the comma. We could not see any other inconsistencies of this type.

***RBC 10:*** In the sentence "The mean completion time for the survey was 30 minutes).", a parenthesis is superfluous.

***ARRB 10:*** The superfluous parenthesis has been removed.
